# Functional and sequence-based metagenomics to uncover carbohydrate-degrading enzymes from composting samples

**DOI:** 10.1007/s00253-023-12627-9

**Published:** 2023-07-07

**Authors:** Cátia Santos-Pereira, Joana Sousa, Ângela M. A. Costa, Andréia O. Santos, Teresa Rito, Pedro Soares, Ricardo Franco-Duarte, Sara C. Silvério, Lígia R. Rodrigues

**Affiliations:** 1 CEB-Centre of Biological Engineering, Universidade Do Minho, Campus de Gualtar, 4710-057 Braga, Portugal; 2 LABBELS–Associate Laboratory, Guimarães, Braga Portugal; 3 CBMA-Centre of Molecular and Environmental Biology, Department of Biology, University of Minho, Campus de Gualtar, 4710-057 Braga, Portugal; 4 IB-S-Institute of Science and Innovation for Bio-Sustainability, University of Minho, Campus de Gualtar, 4710-057 Braga, Portugal

**Keywords:** CAZymes, Composting, Glycoside hydrolases, Lignocellulose, Metagenomics

## Abstract

**Abstract:**

The renewable, abundant , and low-cost nature of lignocellulosic biomass can play an important role in the sustainable production of bioenergy and several added-value bioproducts, thus providing alternative solutions to counteract the global energetic and industrial demands. The efficient conversion of lignocellulosic biomass greatly relies on the catalytic activity of carbohydrate-active enzymes (CAZymes). Finding novel and robust biocatalysts, capable of being active under harsh industrial conditions, is thus imperative to achieve an economically feasible process. In this study, thermophilic compost samples from three Portuguese companies were collected, and their metagenomic DNA was extracted and sequenced through shotgun sequencing. A novel multi-step bioinformatic pipeline was developed to find CAZymes and characterize the taxonomic and functional profiles of the microbial communities, using both reads and metagenome-assembled genomes (MAGs) as input. The samples’ microbiome was dominated by bacteria, where the classes *Gammaproteobacteria*, *Alphaproteobacteria*, and *Balneolia* stood out for their higher abundance, indicating that the degradation of compost biomass is mainly driven by bacterial enzymatic activity. Furthermore, the functional studies revealed that our samples are a rich reservoir of glycoside hydrolases (GH), particularly of GH5 and GH9 cellulases, and GH3 oligosaccharide-degrading enzymes. We further constructed metagenomic fosmid libraries with the compost DNA and demonstrated that a great number of clones exhibited β-glucosidase activity. The comparison of our samples with others from the literature showed that, independently of the composition and process conditions, composting is an excellent source of lignocellulose-degrading enzymes. To the best of our knowledge, this is the first comparative study on the CAZyme abundance and taxonomic/functional profiles of Portuguese compost samples.

**Key points:**

*• Sequence- and function-based metagenomics were used to find CAZymes in compost samples.*

*• Thermophilic composts proved to be rich in bacterial GH3, GH5, and GH9 enzymes.*

*• Compost-derived fosmid libraries are enriched in clones with β-glucosidase activity.*

**Supplementary information:**

The online version contains supplementary material available at 10.1007/s00253-023-12627-9.

## Introduction

The environmental and human health concerns have compelled the replacement of fossil fuels with innovative and sustainable biobased supply chains ensuring economic viability and ecological compatibility. The biorefineries emerge as sustainable and green industrial processes aimed at the effective exploitation of residual biomass resources into bioenergy (biofuels, electricity, and heat) and other value-added products (biopolymers, biochemicals, biofertilizers, or bioplastics) (Duan et al. 2022). It is expected that the biobased product market will reach 50 billion euro by 2030 (Hassan et al. 2019).

Hence, lignocellulosic biomass, being a renewable, low cost, and highly abundant source of organic carbon, has the potential to become one of the world’s leading primary energy sources over the next century, also considering its annual global production (2 × 10^11^ t) (Devi et al. 2022). Lignocellulosic materials comprise food crops (e.g., sugarcane, corn, and soybean), non-food crops (e.g., eucalyptus, grasses, and willow), agroforestry residues (e.g., forest thinning, sugarcane bagasse, and wheat straw), agro-industrial wastes (spent coffee grounds, apple pomace, and municipal sludge), and inedible marine biomass (seaweeds and microalgae) (Hassan et al. 2019; Usmani et al. 2021). Although lignocellulosic residues differ between species, their main organizational components are cellulose (35 to 50%), hemicellulose (20 to 35%), and lignin (5 to 30%), which are arranged in a complex and rigid three-dimensional structure. The conversion of biomass into value-added products includes the following fundamental steps: (1) pretreatment to ease the access to the raw material (e.g., biological, chemical, and physical methods); (2) saccharification through enzymatic hydrolysis; (3) fermentation of sugars; and (4) purification of the end products (Ali et al. 2020). A set of diverse enzymes (carbohydrate-active enzymes, CAZymes) that are categorized into distinct classes and families are required to perform efficient lignocellulose depolymerization. These key enzymes can be classified into the glycoside hydrolases (GHs), auxiliary activities (AAs), glycosyltransferases (GTs), polysaccharide lyases (PLs), and carbohydrate esterases (CEs). Furthermore, useful carbohydrate-binding modules (CBMs) can also be associated to CAZymes (Cantarel et al. 2009; Braga et al. 2021).

There is an increasing need to find novel and robust enzymes with promising features that compete with those currently available on the market. The use of prominent techniques, which are independent of microbial cultivation, namely, metagenomics, allows to explore the genetic and metabolic diversity of complex ecosystems (Datta et al. 2020). Metagenomic studies are based on two main approaches: sequence-based and function-based metagenomics. The sequence-based approach is suitable to find new gene sequences that reveal similarities with the annotated genomes available in the databases. The functional approach allows the identification of unknown genes encoding novel bioactive molecules whose functions/activities would not be predicted only based on the DNA sequence (DeCastro et al. 2016; Madhavan et al. 2017; Datta et al. 2020).

Environmental DNA from soil, water, animal gut, industrial sewages, and extreme environments, such as composting, has been shown to have a great potential to uncover promising enzymes for biotechnological applications (Datta et al. 2020; Sousa et al. 2022). Due to the huge variety of microorganisms able to degrade lignocellulosic biomass involved in composting, it is considered one of the most important (bio)reactors that contribute to the renewable bioenergy on the planet. Some raw materials used in the composting process are green, agricultural, and agro-industrial residues, including animal feces, fruit, vegetable and crop harvesting residues, and municipal wastes. The whole composting process involves four phases, namely, the mesophilic phase, thermophilic phase, cooling phase, and maturation phase. The thermophilic phase, where temperatures above 65 °C can be reached, promotes the proliferation of thermophilic microorganisms able to produce interesting enzymes that degrade cellulose, hemicellulose, lignin, and proteins (Sánchez et al. 2017; Finore et al. 2023).

In this work, a sequence-based and functional metagenomic approach was implemented to identify, for the first time, carbohydrate-degrading enzymes in thermophilic compost samples containing lignocellulosic residues collected from three Portuguese composting units. To achieve this goal and also to obtain the taxonomic and functional profiles of the microbial communities, a multi-step bioinformatic pipeline herein developed was used. Additionally, the presence of β-glucosidase positive clones in the fosmid metagenomic libraries prepared with the DNA extracted from compost samples was experimentally validated. The clones herein described may thus provide excellent alternative biocatalysts that can be explored for a more efficient bioconversion of lignocellulosic biomass towards several industrial applications.

## Materials and methods

### Reagents

Cellulase from *Trichoderma reesei*, lipase from *Candida rugosa*, and all chemicals used in this work (analytical grade) were purchased from Sigma-Aldrich (St. Louis, USA). Proteinase K and RNase A were obtained from NZYTech (Lisbon, Portugal). Commercial humic acids and esculin were supplied by Alfa Aesar (Kandel, Germany). The CopyControl™ fosmid library kit, including the autoinduction solution, was provided by Epicentre Biotechnologies (Madison, Wisconsin, USA).

### Sample collection and characterization

Compost samples with 1–2 kg were obtained in the thermophilic phase from three Portuguese composting companies, namely, Terra Fértil (TF), Lipor (LP), and Gintegral (GN) (Table 1). The sample from TF was collected in the unit of Parque Eco do Relvão, Chamusca (39° 24′ 11.4″ N 8° 23′ 11.9″ W). This composting unit mainly handles municipal sludge and agroforestry residues. The sample from LP was collected in Baguim do Monte, Porto (41° 11′ 58.4″ N 8° 32′ 46.6″ W). Lipor is the intermunicipal waste management service of Porto Metropolitan Area, and it works with food wastes from restaurants and local marketplaces and also green wastes from forests and public gardens. The sample from GN was obtained in the unit of Vitorino das Donas, Ponte de Lima (41° 43′ 17.3″ N 8° 39′ 35.3″ W). This composting unit mainly handles municipal sludge and several organic wastes considered unfit for direct application in agriculture. All the compost samples were stored at 4 °C until physicochemical characterization and DNA extraction.

**Table 1:** Characterization of the compost samples obtained from Terra Fértil, Lipor, and Gintegral. Different superscript letters in the same row indicate significant statistical difference (*p* < 0.05)

	Terra Fértil (TF)	Lipor (LP)	Gintegral (GN)
Date of sampling	October 2019	July 2020	October 2020
Composition (%)	Agroforestry residues (50) and municipal sludge (50)	Food wastes (40), green (25), and forestry (35) residues	Municipal sludge (60), wood and cork chips (30), ashes and fine biomass (10)
Composting period (weeks)	3–4	4	2–3
Depth (cm)	50–60	50	50
Temperature (°C)	62.7 ± 1.0	50.0 ± 1.0	52.0 ± 1.0
Moisture (%)	69.02 ± 2.38^a^	59.55 ± 1.93^b^	63.44 ± 2.20^a,b^
pH	6.96 ± 0.02^a^	8.92 ± 0.08^b^	6.80 ± 0.01^c^
Total carbon (%)	13.23 ± 0.41^a^	17.97 ± 0.04^b^	20.33 ± 3.66^a,b^
Total nitrogen (%)	1.15 ± 0.04^a^	1.66 ± 0.08^b^	1.81 ± 0.35^a,b^
Total hydrogen (%)	4.22 ± 0.21^a^	6.56 ± 0.15^b^	9.56 ± 1.80^a,b^
Total sulfur (%)	0.41 ± 0.01^a^	0.13 ± 0.01^b^	0.24 ± 0.08^a,b^
C:N ratio	11.51 ± 0.07^a^	10.87 ± 0.54^a^	11.25 ± 0.18^a^
Humic acids (mg∙g^−1^ compost)	10.63 ± 0.38^a^	18.42 ± 0.11^b^	6.62 ± 0.21^c^

The compost samples were characterized regarding their temperature, moisture, pH, elemental content, and humic acid concentration, as previously described by Costa et al. (2021). Temperature was determined on site with a thermometer. Briefly, to determine the moisture content, the compost samples were placed at 105 °C until reaching a constant weight. To measure the compost sample pH, a mixture of 4:1 (w/w) of water and compost was prepared, stirred for 30 min, and centrifuged to remove the non-soluble fraction, and the pH of the supernatant was then measured. The elemental analysis (total organic carbon, nitrogen, hydrogen, and sulfur) was performed by automated dry combustion (Requimte/LAQV, Faculty of Sciences and Technology, Nova University of Lisbon, Portugal). The concentration of humic acids was determined by absorbance measurement at 340 nm, using a standard curve (0–500 ng/μl) previously prepared with commercial humic acids, as described by Costa et al. (2021). Briefly, 1 g of compost samples was added to a 0.1 M NaOH solution (9 ml), stirred for 3 h at room temperature, and then centrifuged at 2500 × g for 10 min. The supernatant was acidified to pH 1.0 with HCl and incubated overnight in the dark (room temperature). The humic acids were then obtained by centrifugation (2500 × g, 10 min), air-dried, and resuspended in Tris–EDTA (TE) buffer. In all cases, the analyses were performed in triplicate. The data are presented as the means and respective standard deviations. GraphPad Prism (version 8.0.1.; https://www.graphpad.com/) was used to perform unpaired *t*-test. The same letters represent no significant differences for a 95% confidence level.

### DNA extraction and metagenomic sequencing

The total metagenomic DNA was extracted from the compost samples using a methodology previously established by our group (Costa et al. 2021). This methodology is composed of three main steps, namely, the cell lysis and humic acid removal, the DNA recovery, and the DNA purification. Briefly, the compost samples (1 g) were firstly mixed with 5 ml of optimized lysis buffer containing 100 mM Tris-HCl, 100 mM Na EDTA, 1.5 M NaCl, 100 mM Na_2_HPO_4_, 100 mM CaCl_2_.2H_2_O, 1 mg/ml of proteinase K, 1 mg/ml of lysozyme, 0.2 mg/ml RNase A, 1% (w/v) powdered activated charcoal (4–8 mesh), and 1 g of glass beads (425–600 μm). After mixing using a vortex at maximum speed for 5 min, the samples were incubated for 30 min at 37 °C, 150 rpm. Then, 1 ml of 20% (w/v) sodium dodecyl sulfate was added, and samples were next incubated for 30 min at 65 °C. In a second step, the samples were centrifuged, and the supernatants were gently mixed with 1 volume of chloroform to isopropanol (C to I) (24:1 v/v). After centrifugation, the aqueous phase was used for DNA precipitation by the addition of 1 volume of C_2_H_3_NaO_2_ (3 M, pH 5.2) and 0.4 volumes of 30% (w/v) polyethylene glycol (MW-8000). DNA samples were initially kept at − 20 °C for 20 min and then slowly thawed on ice. The DNA pellet was precipitated by centrifugation and resuspended with 500 μl of TE buffer (10 mM Tris, 1 mM Na EDTA, pH 8.0). Finally, 1 volume of C to I (24:1 v/v) was added to the DNA and the mixture was centrifuged. The aqueous phase was transferred to a new tube followed by the addition of 1 volume of cold isopropanol. This mixture was then incubated for 5 min at 4 °C. The DNA was pelleted by centrifugation and washed twice with 500 μl of 70% (v/v) ethanol. The DNA samples were next centrifuged, and the pellet was air-dried for 10 min at room temperature. The metagenomic DNA samples were then dissolved in 100 μl of TE buffer and stored at 4 °C.

Metagenome analysis by shotgun sequencing was performed by Novogene (Cambridge, United Kingdom) using the Illumina NovaSeq6000 platform (Illumina, San Diego, CA, USA). The raw sequence data were deposited in the NCBI Sequence Read Archive (SRA) database under the Bioproject PRJNA944686, with the following accession numbers: SAMN33758834 (TF sample), SAMN33758835 (GN sample), and SAMN33758836 (LP sample).

### Metagenomic data analysis

**Fig. 1:**
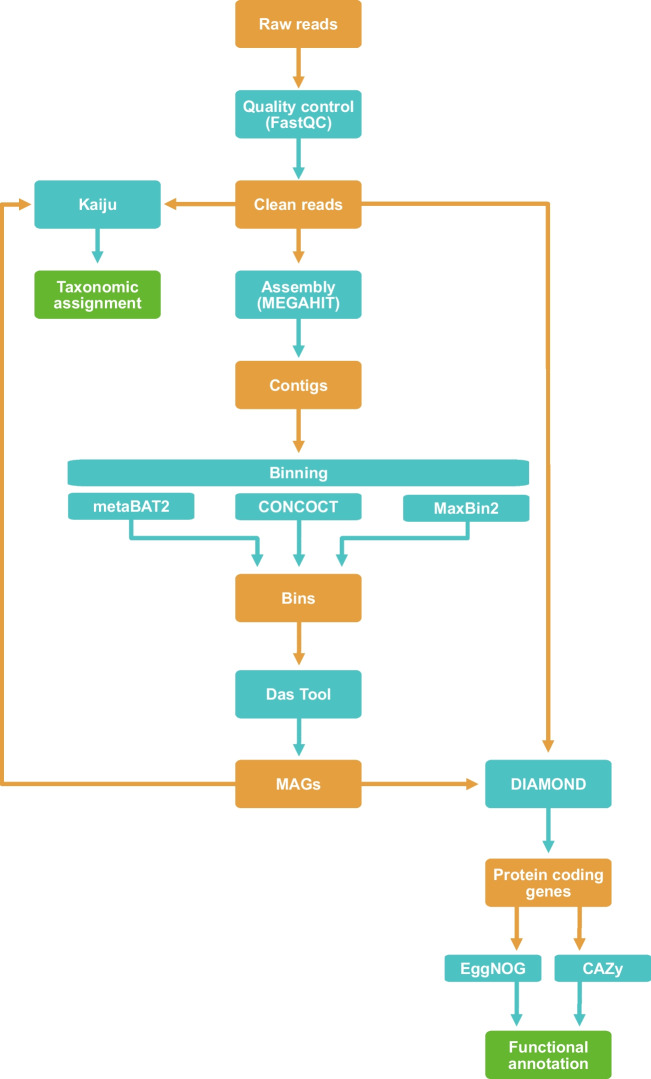
Metagenomics pipeline applied in this study. Workflow of taxonomic classification, genome assembly, binning, gene calling, and functional annotation

The bioinformatic metagenomic pipeline used in this study is depicted in Fig. 1. Throughout the bioinformatic pipeline, different software and parameters were tested at each step, and those rendering more reliable and consistent output were selected to be used in the following steps of the pipeline. Raw sequencing data (raw reads) were checked for quality using FastQC software v. 0. 11.9 (https://www.bioinformatics.babraham.ac.uk/projects/fastqc/). Clean reads were then used to perform taxonomic profiling with Kaiju v.1.9.0 (Menzel et al. 2016), using the nr_euk database (71 GB; 2022-03-10; link: https://kaiju.binf.ku.dk/server), which contains non-redundant proteins belonging to bacteria, archaea, and viruses, but also eukaryotic microorganisms and fungi, covering the most relevant taxonomic groups. Kaiju2table script was used to convert Kaiju’s output files into a summary table for taxonomic ranks.

Clean reads were then assembled into contigs using MEGAHIT v.1.2.9 (Li et al. 2015) and SPAdes v.3.15.4 (Bankevich et al. 2012). Following assembly quality assessment using QUAST software v.5.2.0 (Gurevich et al. 2013), where different parameters including assembly length, contig number, the largest contig, and N50 were evaluated, results from MEGAHIT were chosen for the subsequent analysis. Next, assembled contigs were grouped into bins, using three different binning tools: MaxBin2 (Wu et al. 2016), CONCOCT (Alneberg et al. 2014), and MetaBAT2 (Kang et al. 2019). Afterwards, Das Tool (Sieber et al. 2018) was employed to obtain the metagenome-assembled genomes (MAGs) from the combination of bins obtained by the three tools. For this purpose, only bins with a completeness above 50% and redundancy below 10% were considered. The taxonomic assignment of MAGs was also performed with Kaiju v.1.9.0 software.

Gene calling was performed in two rounds, considering MAGs obtained from Das Tool (Sieber et al. 2018) and also the clean reads. Two software were also used for gene calling—MetaGeneMark v. 3.25 (Zhu et al. 2010) and Diamond v.2.0.15 (Buchfink et al. 2015). A larger number of protein coding genes were obtained with Diamond, so these were used in the subsequent analysis.

Finally, functional annotation of protein coding genes was carried out using eggNOG v.2.1.6 (Jensen et al. 2008) and the CAZy database (Cantarel et al. 2009). Circos plots were created using Circos software v. 0.69.9 (Krzywinski et al. 2009).

### Secondary data analysis

To perform a comparative analysis of our results with metagenomic data from diverse compost samples previously explored, different studies were chosen from the literature. This selection was based on the type of raw materials (mainly lignocellulosic residues), the composting process (homemade composting under uncontrolled conditions or handled composting with controlled environmental conditions), the time of sample collection (during the mesophilic or thermophilic phase of the composting process), and the performance of additional steps, such as enrichment with specific substrates of the compost samples. The exact same pipeline described in Fig. 1 was employed to analyze the reported data, with the exception of the omission of the binning step, since in this case, only the entire set of metagenomic contigs were used.

### Construction of metagenomic fosmid libraries

The metagenomic DNA isolated from the three compost samples (TF, LP, and GN) was directly used in the preparation of three metagenomic libraries using the CopyControl™ fosmid library production kit (Biosearch Technologies, Hoddesdon, UK) and following the manufacturer’s instructions. Briefly, for each metagenomic library, the DNA fragment was end-repaired to generate blunt-ended, 5′-phosphorylated DNA and then, the size of the end-repaired DNA (approximately 40 kb) was confirmed by conventional agarose electrophoresis. Subsequently, the insert DNA was ligated with the linearized and dephosphorylated pCC1FOS™ vector, and the ligation product was packaged into lambda phage. Before plating the metagenomic libraries, the titer of the packaged phage particles was determined to obtain the desired number and density of clones in each plate (around 100 clones). Serial dilutions of packaged phage particles were prepared in phage dilution buffer (PDB) (10 mM Tris-HCl pH 8.3, 100 mM NaCl, 10 mM MgCl_2_) and infected phage T1–resistant EPI300 T1^R^ *Escherichia coli* cells (*trfA* gene, required for replication initiation, was engineered to be under tight control of an inducible promoter) were spread on lysogeny broth (LB) agar plates supplemented with 12.5 μg/ml chloramphenicol and grown overnight at 37 °C. Five hundred sixty-three clones from each metagenomic library were isolated and grown in LB medium supplemented with 10 mM MgSO_4_, 2 g/l maltose, 12.5 μg/ml chloramphenicol, and 0.2% (v/v) CopyControl™ fosmid autoinduction solution (500 ×). Each metagenomic library was stored at − 80 °C in supplemented LB medium with 20% of glycerol.

### β-Glucosidase activity screening and comparison with CAZy prediction

The functional screening of the three metagenomic libraries to detect the β-glucosidase activity was based on a practical and rapid assay performed on 96-well microplates containing a suitable substrate (esculin) for this enzymatic activity.

After the growth of the 563 clones in LB medium supplemented with 12.5 μg/ml chloramphenicol, 10 mM MgSO_4_, 2 g/l maltose, and 0.2% (v/v) CopyControl™ fosmid autoinduction solution (500 ×), they were transferred to 96-well microplates with LB agar, 12.5 μg/ml chloramphenicol, 10 g/l arabinose, 0.1 g/l esculin, and 0.5 g/l ferric chloride. After incubation at 37 °C for 24 h, the 96-well microplates were kept at room temperature (approximately, 25 °C) for 1 week. A color change of the agar culture medium to brown was considered a positive response. Commercial enzymes, namely, cellulase from *T. reesei* and lipase from *C. rugosa*, were used as positive and negative controls, respectively.

In order to compare the results predicted through the CAZy database with the data obtained in the functional screening for β-glucosidase activity, the number of positive clones obtained in the experimental screening after 1 week was compared with the number of predicted CAZymes belonging to the GH1, GH3, GH5, GH9, and GH30 families, which are known to contain β-glucosidases (Cairns and Esen 2010).

## Results

### Physicochemical characterization of the composting samples

Table 1 presents information about the three compost samples collected from TF, LP,, and GN, together with the results obtained for their physicochemical characterization.

As shown in Table 1, three compost samples were obtained from different composting units, which used mixtures of distinct types of input wastes in different proportions. In general, all samples were composed of agroforestry residues or derive from them, and the compost samples from TF and GN contained a significant percentage of municipal sludges. Additionally, the samples from LP and GN presented similar sampling temperatures, while for the TF compost, a higher temperature was recorded (62.7 ± 1.0 °C). Nevertheless, all samples were collected in the thermophilic phase (temperature > 45 °C) of the composting process. Regarding the moisture content, the LP sample presented the lowest value (59.55 ± 1.93%), although not statistically different from the GN sample (63.44 ± 2.20%). In contrast to this parameter, significant statistical differences were obtained for the pH, being the highest value determined for the LP sample (8.92 ± 0.08). For the elemental analysis, significant statistical differences were only found for the TF and LP samples. In all samples, a higher percentage of total carbon (between 13 and 20%) and a lower fraction of total sulfur (< 0.5%) were observed. The total nitrogen values were in all cases in the range of 1–2%. The total hydrogen percentage was lower for the TF compost (4.22 ± 0.21%) and higher for the GN compost (9.56 ± 1.80%). A C to N ratio of approximately 11 was observed for the three samples. Finally, the humic acid content obtained from these samples was statistically different, and the highest value (18.42 ± 0.11 mg∙g^−1^ compost) was determined for the LP sample.

### General features of the metagenome

Metagenome sequencing on the Illumina NovaSeq6000 platform yielded 89.8, 79.9, and 96.3 million raw reads for the TF, LP, and GN samples, respectively, with 150 bp each (Table 2). After the de novo assembly using MEGAHIT, the TF metagenome was found to be composed of 349,015 contigs (186,612 of them with more than 500 bp and the longest having 702,260 bp), with an average GC content of 55.6%, a N50 value of 3990 bp, and with 464 scaffolds higher than 5000 bp. The LP metagenome has 1,055,919 contigs (536,996 of them with more than 500 bp and the longest with 916,338 bp), average GC content of 55.9%, N50 of 1919 bp, and 425 scaffolds higher than 5000 bp. As for the GN metagenome, it has 1,027,319 contigs (530,791 of them with more than 500 bp, contigs, having the longest one 1833 bp), an average GC content of 56.8%, a N50 of 1991 bp, and 663 scaffolds higher than 5000 bp. About 40, 34, and 35% of the assembled contigs can be recruited to the contigs greater than 1000 bp for the TF, LP, and GN samples, respectively. Gene calling based on the assembled contigs using DIAMOND algorithm predicted 677,723 protein coding sequences (CDSs) for the TF sample and more than 1.7 million CDSs for the LP and GN samples, with an average length of 188.2, 168.7, and 170.5 amino acids, respectively. A total of 60.2 and 82.7% of the TF contigs were annotated by the COG (Tatusov et al. 2000) and KEGG (Kanehisa and Goto 2000) databases, respectively. For the LP and GN samples, these percentages were 62.1 and 83.1% (LP) and 64.4 and 61.6% (GN), respectively. Importantly, in the assembled metagenomic contigs of the three samples, 0.91–1.1% were annotated as CAZymes (CAZy database (Cantarel et al. 2009)).

**Table 2:** Summary of sequencing reads, assembly, and gene calling statistics

	Terra Fértil (TF)	Lipor (LP)	Gintegral (GN)
Raw reads	89,773,808	79,895,850	96,255,386
Filtered reads	85,383,033	63,942,544	76,847,071
Number of unmapped reads	4,390,775	15,953,306	19,408,315
Assembly length (bp)	428,104,317	971,450,075	973,097,280
Number of contigs (> 0 bp)	349,015	1,055,919	1,027,319
Number of contigs (> 500 bp)	186,612	536,996	530,791
Largest contig (bp)	702,260	916,338	790,544
Average contig length (bp)	2,294.1	1809	1833
N50 (bp)	3990	1919	1991
L50 (bp)	13,427	71,152	67,169
Number of Ns per 100 kb	0	0	0
GC content (%)	55.55	55.94	56.83
Number of scaffolds > 5000 bp	464	425	663
Total length > 5000 bp	46,317,679	36,018,735	63,399,047
Number of coding genes (CDGs)	677,723	1,743,004	1,702,458
CDGs mapped to COG	408,341	1,081,876	1,096,469
CDGs mapped to KEGG	560,201	1,449,616	1,048,571
CDGs mapped to CAZy	6172	19,001	16,474

### Taxonomic composition of compost microbiota

**Fig. 2:**
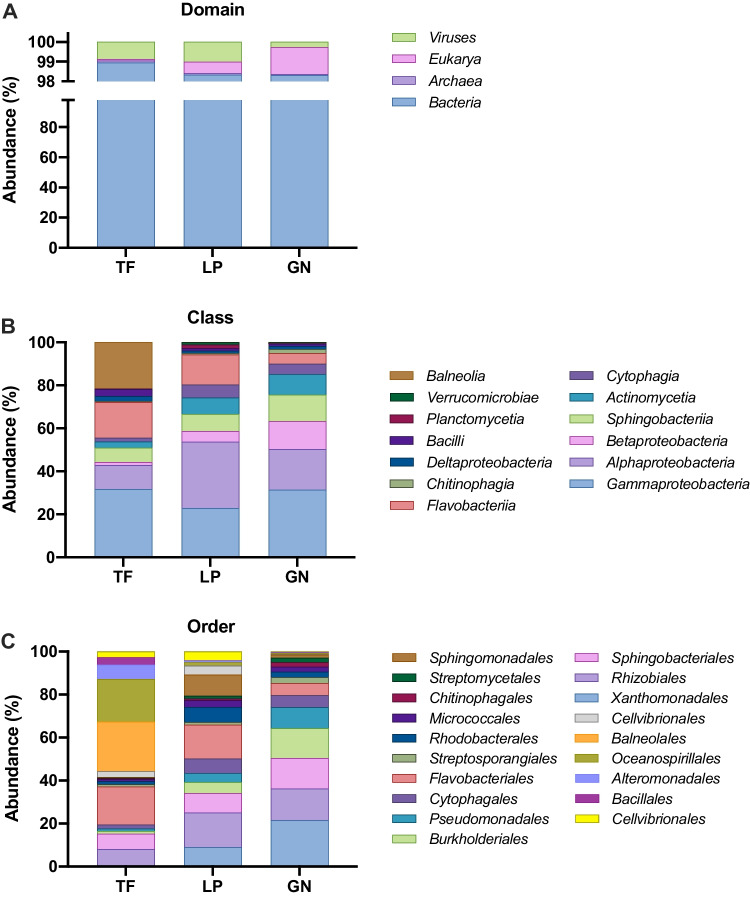
Taxonomic overview of the samples Terra Fértil (TF), Lipor (LP), and Gintegral (GN). Taxonomic distribution of the metagenomes based on relative abundances of the clean metagenomic reads at domain (**A**), class (**B**), and order (**C**) levels

The taxonomic classification of the metagenomic samples was performed with the clean reads (prior to assembly) using the Kaiju software (Menzel et al. 2016) and the nr_euk database. For the three samples, around 70% of the reads could be assigned to a taxon, leaving only around 30% that could not be assigned to any community, which validates the use of the software and of the database. Results from domain analysis (Fig. 2A) showed that all samples were clearly dominated by *Bacteria* (98.3–98.9%). In the TF sample, this percentage was followed by *Viruses* (0.9%), *Archaea* (0.1%), and *Eukaryota* (0.07%). As for the LP sample, *Bacteria* higher abundance was followed by *Viruses* (1%), *Eukaryota* (0.6%), and *Archaea* (0.07%). Finally, in the GN sample, *Eukaryota* (1.4%) followed *Bacteria* in terms of relative abundance and then *Viruses* (0.3%) and *Archaea* (0.05%). The taxonomic analysis also revealed a similar number of species, genera, families, orders, and classes among the three samples, with mean values of 32,740 species; 4189 genera; 1019 families; 473 orders; and 200 classes (Supplemental Tables S1–S6).

In terms of class taxonomic assessment (Fig. 2B), the TF sample was dominated by *Gammaproteobacteria* (31.6%) and *Balneolia* (21.6%), with these two classes accounting for more than 50% of the community and being this high amount of *Balneolia* bacteria exclusive of this sample. *Flavobacteriia* (16.6%) and *Alphaproteobacteria* (11.3%) were the next two most representative classes in this sample. The predominant class in the LP sample was *Alphaproteobacteria* (30.9%), which was also only observed for this sample, followed by *Gammaproteobacteria* (22.8%) and *Flavobacteriia* (14%). Regarding the GN sample, the class *Gammaproteobacteria* (31.4%) exhibited the highest relative abundance, as it was also detected in the TF sample, followed by *Alphaproteobacteria* (19%), *Betaproteobacteria* (12.9%) and *Sphingobacteriia* (12.4%). Regarding the order of taxonomic identification, a very heterogenous pattern was observed between samples. The most abundant orders (Fig. 2C) were *Balneolales* (23.1%) in the TF sample, *Rhizobiales* (16.2%) and *Flavobacteriales* (15.7%) in the LP sample, and *Xanthomonadales* (21.5%) in the GN sample.

### Functional profiles and metabolic pathway annotation of the metagenomes

**Fig. 3:**
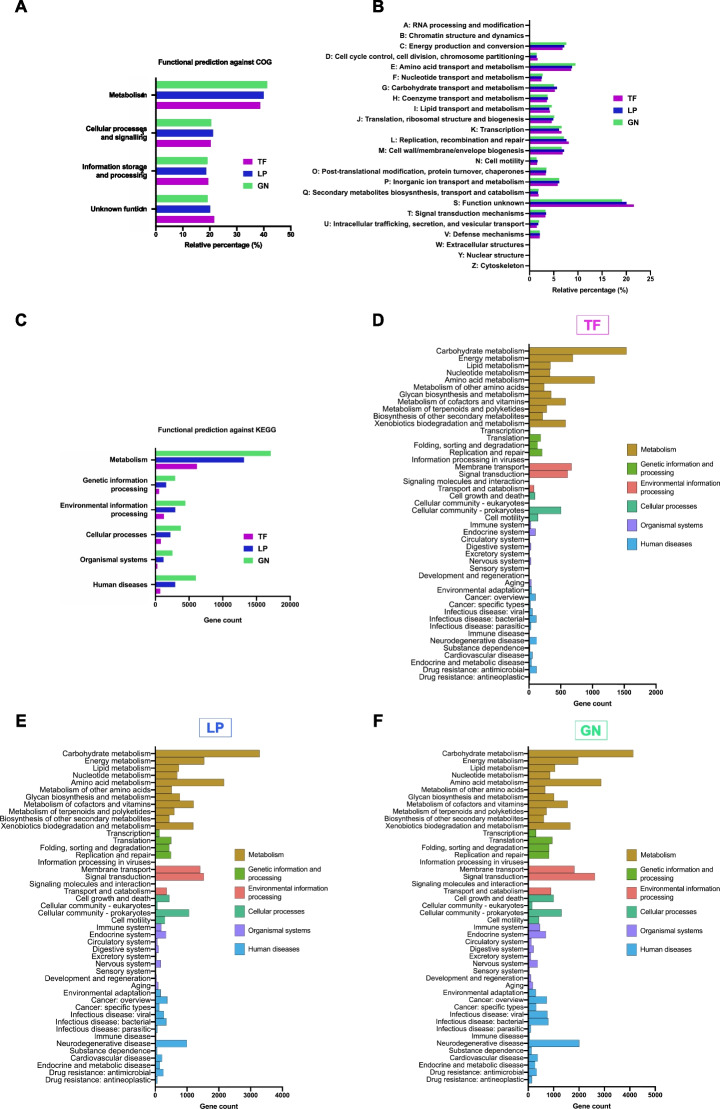
Functional and metabolic classification based on COG and KEGG databases for Terra Fértil (TF), Lipor (LP) and Gintegral (GN) samples. (**A**) Distribution of the main categories of the COG database across the three samples. (**B**) Distribution of COG sub-categories across the three samples. (**C**) Distribution of genes among level 1 KEGG metabolic pathways for the TF, LP and GN samples. Distribution of genes among level 2 KEGG metabolic pathways for the TF (**D**), LP (**E**) and GN (**F**) samples.

To analyze the metagenomes’ functional profiles, after assembly, the putative protein coding genes identified using DIAMOND algorithm were mapped against the COG database (Tatusov et al. 2000). In all three samples, the most predominant functional category was “metabolism” followed by an identical percentage of sequences belonging to the categories “cellular processes and signalling” and “information storage and processing” (Fig. 3). Only around 20% of the sequences were assigned to “unknown function” in all samples (Fig. 3A). Within the “metabolism” category, the most abundant COG categories for all samples were “amino acid transport and metabolism,” followed by “energy production and conversion,” “inorganic ion transport and metabolism,” “carbohydrate transport and metabolism,” and “lipid transport and metabolism.” Within the “cellular processes and signalling” and “information storage and processing” categories, the most abundant COG categories were “cell wall/membrane/envelope biosynthesis” and “replication, recombination and repair,” respectively (Fig. 3B). In terms of sample comparison, no large differences were detected between samples.

Annotation of the metagenomic contigs against the KEGG database (Kanehisa and Goto 2000) was performed to identify the putative genes mapped to different metabolic pathways. In agreement with the annotation against the COG database, pathways related to “metabolism” were by far the most abundant among the three samples, with the LP and GN samples displaying a much higher gene count within this category than the TF sample (Fig. 3C). The GN sample showed the highest number of genes associated with “metabolism,” being this number more than three times higher than the one found in the TF sample. Additionally, the “carbohydrate metabolism”–related pathways were those gathering a higher number of putative genes in all samples, followed by “amino acid metabolism,” “energy metabolism,” “metabolism of cofactors and vitamins,” and “xenobiotics biodegradation and metabolism.” Within the other level 2 KEGG pathways, a high number of genes were mapped to the pathways associated with “membrane transport” and “signal transduction” for all samples and “neurodegenerative diseases” for the GN and LP samples (Fig. 3D).

The combined analysis of functional annotation against KEGG and COG databases revealed a clear dominance of the contigs associated with metabolism on the compost metagenomic samples, with particular emphasis on carbohydrate metabolism.

### Mining of carbohydrate-active enzymes

In order to identify putative enzymes able to degrade lignocellulosic biomass in the metagenomes, the reads were queried against the CAZy database (Cantarel et al. 2009; Drula et al. 2022), which is dedicated to enzymes involved in the building and breakdown of complex carbohydrates (CAZymes). Among the protein coding genes, around 6170; 19,000; and 16,470 for the TF, LP, and GN samples, respectively, were mapped to this database corresponding to approximately 1% of all the predicted genes (Table 2).

**Fig. 4:**
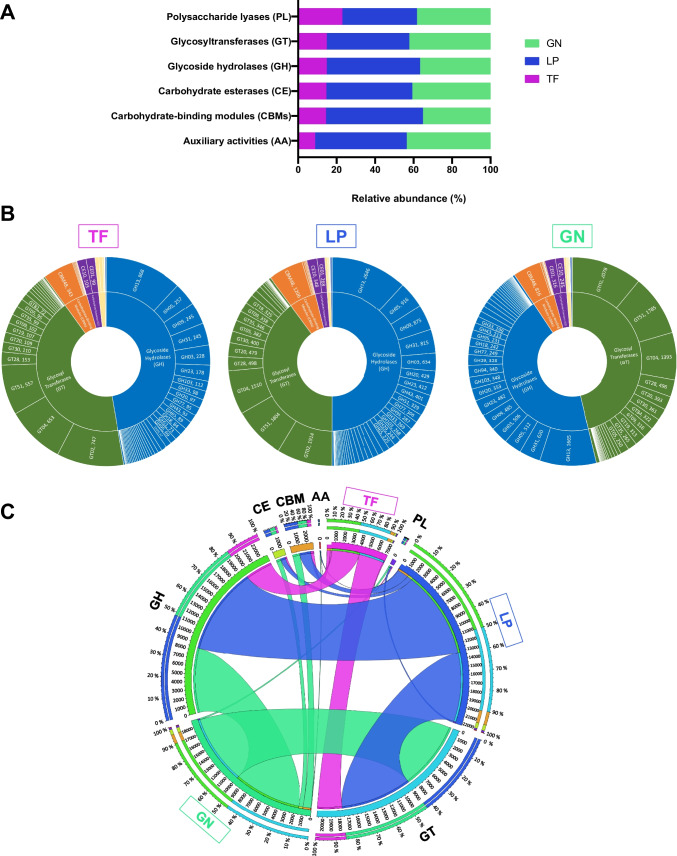
Distribution of carbohydrate-active enzymes (CAZymes) in the Terra Fértil (TF), Lipor (LP), and Gintegral (GN) samples. **A** Contribution of each sample to the total number of protein coding sequences assigned to each CAZyme family.** B** Distribution of CAZymes in the three samples. The inner ring describes the main CAZyme classes (GT, green; GH, dark blue; CBMs, orange; CE, purple; AA, light blue; PL, light yellow), while the outer ring depicts the families, as well as the corresponding number of protein coding sequences annotated. **C** Circos plot depicting the relative abundance of CAZymes in the TF, LP, and GN samples. The inner ring indicates the total number of protein coding sequences associated to each sample or CAZyme class; the outer ring represents the relative abundance of protein coding sequences from each sample or CAZyme class; the width of the bars connecting a given sample and CAZyme class indicates their relative abundance to each other. GH glycoside hydrolases, GT glycosyltransferases, PL polysaccharide lyases, CE carbohydrate esterases, CBMs carbohydrate-binding modules, AA auxiliary activities

The CAZy classification comprises 5 classes of enzymatic activities: GHs that include glycosidases and transglycosidases; GTs which can be either retaining or inverting enzymes; PLs that include enzymes with an ability to cleave uronic acid–containing polysaccharide chains; CEs which catalyze the de-O or de-N-acylation of substituted saccharides; and AAs that describe a wide variety of enzyme mechanisms and substrates associated with lignocellulose conversion including lignin degradation. Additionally, carbohydrate-active enzymes often display non-catalytic modules, the CBMs, which are protein fragments that lack enzymatic activity per se but potentiate the activity of the aforementioned enzymes (Lombard et al. 2014). The 6 enzymatic activities were detected in all samples revealing the potential of the putative genes encoded in the compost metagenomes for lignocellulose degradation. Considering the total number of genes from the three samples assigned to each individual family, it is evident that the LP and GN samples displayed a higher number of CAZymes when compared with the TF sample (Fig. 4A).

Considering the relative abundance of each enzymatic family in the metagenomic samples, clearly, the three samples were dominated by GHs and GTs (Fig. 4B and C). The GT2 family was the most abundant in all composting samples, with GT51 and GT4 also counting with many putative genes from the metagenomes (Fig. 4B). For GHs, the GH13 family was by far the most abundant in all samples, followed by GH3, GH5, GH9, and GH31 (Fig. 4B).

**Fig. 5:**
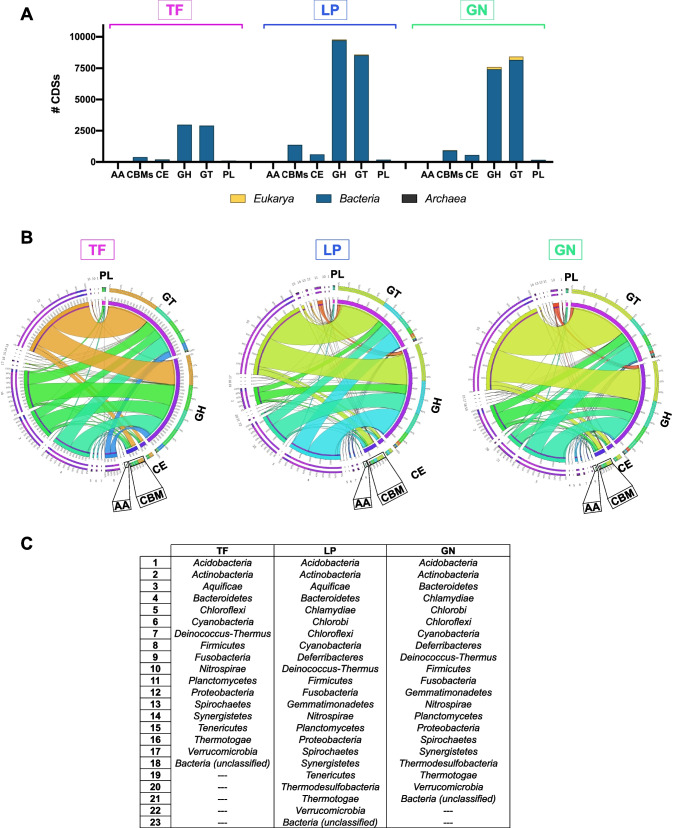
Taxonomic distribution of bacterial CAZymes in the Terra Fértil (TF), Lipor (LP), and Gintegral (GN) samples. **A** Distribution of protein coding sequences (CDSs) annotated to the CAZy database among the three domains of life (*Bacteria*, *Archaea*, and *Eukarya*) in the three compost samples. **B** Circos plots depicting the taxonomic affiliation of CAZymes in the TF, LP, and GN samples. The enzyme classes are on the right side and the phyla numbers on the left; the *inner ring* represents the total number of protein coding sequences associated to each CAZyme class or phylum; the *outer ring* represents the relative abundance of protein coding sequences from each CAZyme class or phylum; the width of the bars connecting a given CAZyme class and phylum indicates their relative abundance with respect to each other. **C** Support table that identifies the phylum each number corresponds in the circos plots depicted in **B** for each sample. GH glycoside hydrolases, GT glycosyltransferases, PL polysaccharide lyases, CE carbohydrate esterases, CBMs carbohydrate-binding modules, AA auxiliary activities

Regarding the taxonomic classification of the putative enzymes found in the metagenomes, CAZymes largely belonged to the *Bacteria* domain (Fig. 5A), being particularly distributed through 17–22 bacterial phyla (Fig. 5B and C). Importantly, in all samples, the four most abundant CAZyme classes (GHs, GTs, CBMs, and CEs) were clearly affiliated to three different bacterial phyla: *Proteobacteria*, *Bacteroidetes*, and *Actinobacteria* (Fig. 5B and C). Though the great majority of CAZymes found in our samples were bacterial, among the domains *Archaea* and *Eukarya*, the phylum *Euryarchaeota* and the kingdom *Fungi* showed higher abundances in both GHs and GTs, respectively, except for the TF sample that had a higher portion of GHs in the *Metazoa* kingdom (Supplemental Fig. S1).

### Lignocellulose degradation potential of MAGs retrieved from compost samples

To identify the microorganisms with higher potential for lignocellulose degradation within the collected compost samples, binned MAGs with completeness ≥ 50% and redundancy < 10% were recovered and further analyzed regarding taxonomy, functionality, and presence of CAZymes. A total of 44, 66, and 69 of MAGs that fulfilled these parameters were recovered from the TF, LP, and GN samples, respectively. The features and statistics of MAG assemblies, as well as the maximum predicted taxonomic level are summarized in Supplemental Table S7. The average size of MAGs was around 3.2 Mb with a mean N50 of 38,900 bp. The GC content varied from 33.2 to 74.4%, indicating a broad range of microorganisms.

**Fig. 6:**
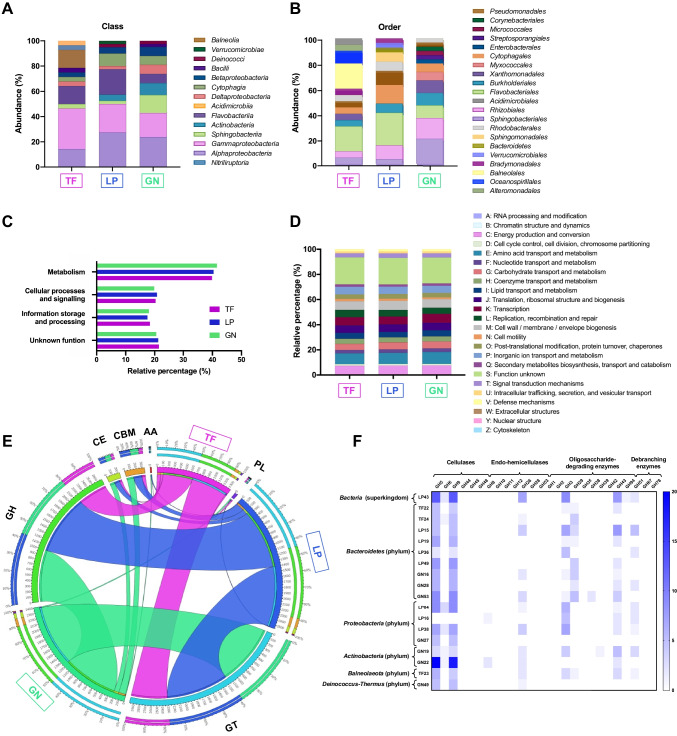
Taxonomic classification, functional annotation, and identification of CAZymes in the metagenome-assembled genomes (MAGs) recovered from Terra Fértil (TF), Lipor (LP), and Gintegral (GN) samples. **A** Taxonomic distribution of MAGs that could be assigned to the class level. Not classified MAGs were excluded from the calculation of abundancy. **B** Taxonomic classification of MAGs that could be affiliated at the order level. Not classified MAGs were excluded from the calculation of abundancy. **C** Functional annotation of MAGs recovered from the three samples based on the main categories of the COG database. **D** Distribution of COG sub-categories across the MAGs recovered from three samples. **E** Circos plot depicting the relative abundance of CAZymes in MAGs recovered from the TF, LP, and GN samples. The *inner ring* indicates the total number of protein coding sequences associated to each sample or CAZyme class; the *outer ring* represents the relative abundance of protein coding sequences from each sample or CAZyme class; the *width* of the bars connecting a given sample and CAZyme class indicates their relative abundance to each other. GH glycoside hydrolases, GT glycosyltransferases, PL polysaccharide lyases, CE carbohydrate esterases, CBMs carbohydrate-binding modules, AA auxiliary activities. **F** Presence of the GH families associated with lignocellulose-degrading enzymes in the MAGs recovered from the three samples. The heatmap represents the number of protein coding sequences from each MAG mapped to each family. Only MAGs displaying at least five protein coding sequences belonging to any GH family were included. The taxonomic identification at the phylum level is also depicted, except for the LP43 to which the maximum taxonomic level obtained was the kingdom

Figure 6A and B shows the taxonomic inference of MAGs at the class and order levels. It is worth mentioning that not all MAGs could be identified at these levels (Supplemental Table S7). Indeed, 40.9–45.5% of MAGs could not be classified at the class level and 60.6–63.6% at the order level. Nevertheless, all the putative genomes were assigned to the *Bacteria* domain (Supplemental Table S7) (Fig. 2A). Taking into account the MAGs classified at the class level, the TF sample was clearly dominated by bacteria belonging to the *Gammaproteobacteria* class, followed by *Alphaproteobacteria*, *Flavobacteriia*, and *Balneolia*. The LP and GN samples had higher percentage of bacteria belonging to *Alphaproteobacteria*, followed by *Gammaproteobacteria*. However, while the third most abundant class in the LP sample was *Flavobacteriia*, in the GN sample, it was *Sphingobacteriia* (Fig. 6A). At the order level, the two most abundant in the TF sample were *Flavobacteriales* and *Balneolales* accounting for nearly 20% of the orders represented in the repertoire of MAGs from this sample. As for the LP sample, it was dominated by *Flavobacteriales* (25.9%) followed by *Cytophagales* (14.8%). Lastly, the GN sample had a higher abundance of *Sphingobacteriales* (20%) followed by *Rhizobiales* (16.7%) (Fig. 6B).

Of note, 9 MAGs could be assigned at the species level. Specifically, in the TF sample, 2 MAGs were affiliated to *Lysobacter defluvii*, 1 MAG to *Pedobacter indicus*, and 1 MAG to *Galbibacter marinus*. In the LP sample, 2 MAGs were assigned to *Bradymonas sediminis*, 1 MAG to *Paracoccus alcaliphilus*, and another one to *Rhodothermus marinus*. In the GN sample, 1 MAG was identified as *Pseudofulvimonas gallinarii* (Supplemental Table S7).

Functionally, the annotation using eggNOG revealed that MAGs collected from the three samples were very similar with the most represented COG category being “metabolism” (Fig. 6C) and with the most abundant sub-categories being “amino acid transport and metabolism,” followed by “energy production and conversion,” “inorganic ion transport and metabolism” and “carbohydrate transport and metabolism” for all samples (Fig. 6D). These data indicate that the differences observed at physicochemical characterization did not affect their overall functional behavior.

To unveil the lignocellulose degradation potential of the MAGs recovered from the compost samples, MAGs were queried against the CAZy database to identify the genes encoding lignocellulolytic enzymes. Results were in agreement with those obtained with clean reads as they showed that, in all samples, the most represented CAZyme families were GHs and GTs (Fig. 6E). Indeed, an average of 25.4 and 16.6 CDSs affiliated to GHs and GTs, respectively, were identified in the compost samples’ MAGs (Supplemental Table S7).

Among CAZymes, GHs are of particular interest as they include a wide range of enzymes useful for the utilization of lignocellulosic biomass (Gong et al. 2020; Reichart et al. 2021). Therefore, the abundance of GH families related to cellulases, endo-hemicellulases, debranching enzymes, and oligosaccharide-degrading enzymes in the retrieved MAGs was studied in detail (Fig. 6F, Supplemental Table S7). Figure 6F shows the MAGs that had at least five CDSs affiliated to any GH family related to the aforementioned categories. It is noteworthy that the LP and GN samples were enriched in MAGs that fulfill these criteria (7–8) in comparison with the TF sample, which was only represented by 3 MAGs. Overall, the genes encoding lignocellulolytic enzymes were distributed through the following families: cellulases (GH5, GH6, GH9), endo-hemicellulases (GH26), oligosaccharide-degrading enzymes (GH3, GH29, GH43), and debranching enzymes (GH51). Among these families, GH5 and GH9 clearly stood out as all the depicted MAGs displayed at least 5 CDSs belonging to these families, except for the LP16, and with particular abundance in the LP43 and GN22 MAGs (Fig. 6F).

Considering the taxonomic affiliation, 50% of the MAGs enriched in lignocellulose-degrading enzymes belonged to the phylum *Bacteroidetes*, followed by *Proteobacteria* (22.2%). Additionally, 2 MAGs assigned to the phylum *Actinobacteria*, 1 to *Balneolaeota*, and 1 to *Deinococcus-Thermus* also showed an interesting abundance of these enzymes. Notably, the *Balneolaeota* TF23 MAG, a phylum that was only present in the TF sample, exhibited GHs belonging to all the main categories represented in the samples from this study. Curiously, there was no clear distribution of a particular GH family across a particular phylum, but some families appeared only in a single MAG. Indeed, the α-mannosidases GH38 were only present in the GN53 MAG (*Bacteroidetes*), the β-xylosidases GH39 in the GN19 MAG (*Actinobacteria*), and the cellobiose phosphorylases GH94 in the LP43 MAG (could only be assigned to the *Bacteria* kingdom). In addition, the endo-1,4-β-xylanases GH8 appeared only in the LP16 (*Proteobacteria*) and GN22 (*Actinobacteria*) MAGs (Fig. 6F).

### Diversity of lignocellulose-degrading glycoside hydrolases in compost metagenomes from diverse origins

To compare the potential for lignocellulosic biomass conversion of the samples herein described with other compost samples holding different features and composition, we next analyzed the abundance of the GH families more important for plant biomass processing (Gong et al. 2020; Reichart et al. 2021) in the TF, LP, and GN samples and also in composting samples selected from the literature. For that purpose, the metagenomic sequencing data from the selected samples were processed using the same pipeline applied in this work (Fig. 1, Supplemental Table S8) and a particular set of genes encoding for four categories of lignocellulose-degrading enzymes, namely, cellulases, endo-hemicellulases, debranching enzymes, and oligosaccharide-degrading enzymes (Table 3), was evaluated, as described by Gong et al. (2020) and Reichart et al. (2021).

**Table 3:** Comparison of the genes encoding glycoside hydrolases (GHs) and respective subfamilies with recognized activity for lignocellulosic biomass degradation in the compost samples described in this study with compost samples from other studies. The general composition of the samples can be found in the second line. The main activity of selected GH subfamilies is on the left column. In bold are the samples exhibiting more than 5% protein coding sequences (#) affiliated to a given subfamily

		Sample	TF	LP	GN	SRR032208 (SG)	SRR10856164 (CBLU)	SRR4381490 (AD)	SRR4381491 (EC)	SRR4381492 (PN)	SRR2388864 (FN1)	SRR2388865 (FN2)	SRR2388866 (FN3)
		Composition	Agroforestry residues and municipal sludge compost	Food wastes, green and forestry residue compost	Municipal sludge, wood and cork chip, ashes, and fine biomass compost	Switchgrass and green-waste compost	Corncob, bran, lime, and urea compost	Chipped wood from *Arundo donax*	Chipped wood from *Eucalyptus camaldulensis*	Chipped wood from *Populus nigra*	Forest compost soil enriched on Napier grass (day 2)	Forest compost soil enriched on Napier grass (day 7)	Forest compost soil enriched on Napier grass (day 19)
		Ref	This study	This study	This study	Allgaier et al. (2010)	Kong et al. (2020)	Montella et al. (2017)	Montella et al. (2017)	Montella et al. (2017)	Kanokratana et al. (2018)	Kanokratana et al. (2018)	Kanokratana et al. (2018)
	# GHs	GHs	3409	11120	8378	1137	4960	8785	10328	1502	1462	1476	1772
	% GH/CAZymes		55.2	58.5	50.9	58.9	49.1	57.8	58.5	58.9	59.9	61.2	62.4
Cellulase	# GH5	Cellulases	**257 (7.5%)**	**916 (8.2%)**	**512 (6.1%)**	**71 (6.2%)**	**312 (6.3%)**	**461 (5.2%)**	**696 (6.7%)**	**77 (5.1%)**	**140 (9.6%)**	**145 (9.8%)**	**159 (9.0%)**
Endoglucanase	# GH6		16 (0.5%)	66 (0.6%)	20 (0.2%)	9 (0.8%)	14 (0.3%)	67 (0.8%)	65 (0.6%)	10 (0.7%)	0 (0.0%)	0 (0.0%)	1 (0.1%)
Endoglucanase	# GH9		**245 (7.2%)**	**875 (7.9%)**	**485 (5.8%)**	**69 (6.1%)**	**291 (5.9%)**	**432 (4.9%)**	**618 (6.0%)**	**75 (5.0%)**	**130 (8.9%)**	**138 (9.3%)**	**156 (8.8%)**
Endoglucanase	# GH44		0 (0.0%)	0 (0.0%)	0 (0.0%)	0 (0.0%)	0 (0.0%)	0 (0.0%)	0 (0.0%)	0 (0.0%)	0 (0.0%)	0 (0.0%)	0 (0.0%)
Endoglucanase	# GH45		0 (0.0%)	0 (0.0%)	0 (0.0%)	0 (0.0%)	0 (0.0%)	0 (0.0%)	0 (0.0%)	0 (0.0%)	0 (0.0%)	0 (0.0%)	0 (0.0%)
Cellobiohydrolase	# GH48		0 (0.0%)	1 (0.0%)	0 (0.0%)	0 (0.0%)	6 (0.1%)	0 (0.0%)	0 (0.0%)	0 (0.0%)	5 (0.3%)	2 (0.1%)	1 (0.1%)
		Total	15.2%	16.7%	12.1%	13.1%	12.0%	10.9%	13.3%	10.8%	18.8%	19.2%	18.0%
Endo-1,4-β-xylanase	# GH8	Endo-hemicellulases	24 (0.7%)	49 (0.4%)	39 (0.5%)	8 (0.7%)	62 (1.3%)	53 (0.6%)	55 (0.5%)	10 (0.7%)	20 (1.4%)	24 (1.6%)	18 (1.0%)
Endo-1,4-β-xylanase	# GH10		0 (0.0%)	0 (0.0%)	0 (0.0%)	0 (0.0%)	0 (0.0%)	0 (0.0%)	0 (0.0%)	0 (0.0%)	0 (0.0%)	0 (0.0%)	0 (0.0%)
Xylanase	# GH11		0 (0.0%)	0 (0.0%)	11 (0.1%)	0 (0.0%)	0 (0.0%)	0 (0.0%)	0 (0.0%)	0 (0.0%)	0 (0.0%)	0 (0.0%)	0 (0.0%)
Xyloglucan endo-hydrolase	# GH12		0 (0.0%)	0 (0.0%)	2 (0.0%)	0 (0.0%)	0 (0.0%)	24 (0.3%)	53 (0.5%)	0 (0.0%)	0 (0.0%)	0 (0.0%)	0 (0.0%)
Xyloglucanase	# GH26		44 (1.3%)	196 (1.8%)	120 (1.4%)	27 (2.4%)	74 (1.5%)	44 (0.5%)	53 (0.5%)	26 (1.7%)	44 (3.0%)	33 (2.2%)	31 (1.7%)
Polygalacturonase	# GH28		9 (0.3%)	1 (0.0%)	3 (0.0%)	1 (0.1%)	22 (0.4%)	14 (0.2%)	26 (0.3%)	0 (0.0%)	0 (0.0%)	0 (0.0%)	0 (0.0%)
Endo-β-1,4-galactanase	# GH53		0 (0.0%)	0 (0.0%)	0 (0.0%)	0 (0.0%)	0 (0.0%)	0 (0.0%)	0 (0.0%)	0 (0.0%)	0 (0.0%)	0 (0.0%)	0 (0.0%)
		Total	2.3%	2.2%	2.0%	3.2%	3.2%	1.6%	1.8%	2.4%	4.4%	3.8%	2.7%
β-Glucosidase	# GH1	Oligosaccharide-degrading enzymes	0 (0.0%)	2 (0.0%)	6 (0.1%)	0 (0.0%)	0 (0.0%)	69 (0.8%)	126 (1.2%)	0 (0.0%)	0 (0.0%)	1 (0.1%)	0 (0.0%)
β-Galactosidase	# GH2		0 (0.0%)	0 (0.0%)	0 (0.0%)	0 (0.0%)	0 (0.0%)	0 (0.0%)	0 (0.0%)	0 (0.0%)	0 (0.0%)	0 (0.0%)	0 (0.0%)
β-Glucosidase	# GH3		**228 (6.7%)**	**634 (5.7%)**	**506 (6.0%)**	**100 (8.8%)**	**479 (9.7%)**	**1160 (13.2%)**	**1636 (15.8%)**	**195 (13.0%)**	**237 (16.2%)**	**231 (15.7%)**	**289 (16.3%)**
α-L-Fucosidase	# GH29		67 (2.0%)	244 (2.2%)	328 (3.9%)	26 (2.3%)	117 (2.4%)	305 (3.5%)	272 (2.6%)	35 (2.3%)	57 (3.9%)	**81 (5.5%)**	**109 (6.2%)**
β-Galactosidase	# GH35		0 (0.0%)	0 (0.0%)	0 (0.0%)	0 (0.0%)	0 (0.0%)	0 (0.0%)	0 (0.0%)	0 (0.0%)	0 (0.0%)	0 (0.0%)	0 (0.0%)
α-Mannosidase	# GH38		63 (1.8%)	158 (1.4%)	125 (1.5%)	17 (1.5%)	106 (2.1%)	0 (0.0%)	264 (2.6%)	56 (3.7%)	53 (3.6%)	54 (3.7%)	47 (2.7%)
β-Xylosidase	# GH39		1 (0.0%)	5 (0.0%)	15 (0.2%)	0 (0.0%)	3 (0.1%)	11 (0.1%)	15 (0.1%)	6 (0.4%)	0 (0.0%)	0 (0.0%)	0 (0.0%)
β-Galactosidase	# GH42		0 (0.0%)	0 (0.0%)	0 (0.0%)	0 (0.0%)	0 (0.0%)	0 (0.0%)	0 (0.0%)	0 (0.0%)	0 (0.0%)	0 (0.0%)	0 (0.0%)
β-Xylosidase	# GH43		93 (2.7%)	401 (3.6%)	213 (2.5%)	46 (4.0%)	**277 (5.6%)**	244 (2.8%)	322 (3.1%)	**82 (5.5%)**	**75 (5.1%)**	71 (4.8%)	80 (4.5%)
Cellobiose phosphorylase	# GH94		28 (0.8%)	220 (2.0%)	340 (4.1%)	10 (0.9%)	76 (1.5%)	277 (3.2%)	194 (1.9%)	42 (2.8%)	26 (1.8%)	22 (1.5%)	16 (0.9%)
		Total	14%	14.9%	18.3%	17.5%	21.4%	23.6%	27.3%	27.7%	30.6%	31.2%	30.6%
α-L-Arabinofuranosidase	# GH51	Debranching enzymes	73 (2.1%)	329 (3.0%)	166 (2.0%)	**68 (6.0%)**	**269 (5.4%)**	269 (3.1%)	336 (3.3%)	67 (4.5%)	57 (3.9%)	56 (3.8%)	67 (3.8%)
α-Glucuronidase	# GH67		0 (0.0%)	0 (0.0%)	0 (0.0%)	0 (0.0%)	0 (0.0%)	0 (0.0%)	0 (0.0%)	0 (0.0%)	0 (0.0%)	0 (0.0%)	0 (0.0%)
α-L-Rhamnosidase	# GH78		0 (0.0%)	0 (0.0%)	0 (0.0%)	0 (0.0%)	0 (0.0%)	0 (0.0%)	0 (0.0%)	0 (0.0%)	0 (0.0%)	0 (0.0%)	0(0.0%)
		Total	2.1%	3.0%	2.0%	6.0%	5.4%	3.1%	3.3%	4.5%	3.9%	3.8%	3.8%

A detailed description of the selected samples can be found in Supplemental Table S9. Sample SRR032208 (SG) was collected from switchgrass feedstock inoculated in bioreactor with green-waste compost (Allgaier et al. 2010); sample SRR10856164 (CBLU) was harvested from a pilot experiment performed in ventilated compartments of compost containing corncob, bran, lime, and urea (Kong et al. 2020); samples SRR4381490 (AD), SRR4381491 (EC) and SRR4381492 (PN) were collected from piles of chipped wood from *Arundo donax*, *Eucalyptus camaldulensis*, and *Populus nigra*, respectively, subjected to natural biodegradation under oak trees (Montella et al. 2017); and finally, samples SRR2388864 (FN1), SRR2388865 (FN2) and SRR2388866 (FN3) were harvested after 2, 7, and 19 days of cultivation, respectively, of a symbiotic cellulolytic microbial consortium based on forest compost soil that was subjected to successive enrichment on Napier grass under facultative anoxic conditions (Kanokratana et al. 2018).

The LP and EC samples were those with higher number of CDSs annotated as GHs (> 10,000) followed by the GN and AD samples (> 8000). Analysis of the abundance of GHs in relation to the total number of CDSs annotated to the CAZy database revealed that all compost samples have a similar percentage of GHs ranging from 49.1% in the CBLU sample to 62.4% in the FN3 sample (Table 3), attesting the widely recognized richness of lignocellulolytic enzymes present in compost samples (Allgaier et al. 2010; Montella et al. 2017; Kanokratana et al. 2018; Kong et al. 2020). The most abundant category in the TF and LP samples was cellulases (15.2 and 16.7%, respectively), while the GN sample from this study and all the samples selected from the literature mainly contained oligosaccharide-degrading enzymes (17.5–31.2%). On the other hand, as above described for MAGs, the most prevalent families of GHs (5.7–8.2%) in our samples were the cellulases GH5, the endoglucanases GH9, and the β-glucosidases GH3. Interestingly, the same families were also among the most represented in the samples selected for comparison (4.9–16.3%). However, in addition to those, the CBLU, PN, and FN1 samples were further enriched in the β-xylosidases belonging to the family GH43 (5.1–5.6%) and the FN2 and FN3 samples in the α-L-fucosidases of the GH29 family (5.5–6.2%). The SG and CBLU samples were also abundant in debranching enzymes with α-L-arabinofuranosidase activity from the GH51 family (5.4–6.0%). Though much less represented, genes encoding for endo-hemicellulases of the GH8, GH26, and GH28 families with endo-1,4-β-xylanase, xyloglucanase, and polygalacturonase activities, respectively, were also found in the selected compost samples (Table 3).

### β-Glucosidase activity in the metagenomes of compost samples

Since GHs are one of the main CAZyme families represented in the compost metagenomes and given that they hold a great potential for lignocellulose biomass degradation, we next decided to construct metagenomic fosmid libraries with the DNA extracted from the three compost samples (TF, LP, and GN), to experimentally validate the presence and abundance of these enzymes in the collected samples. Since β-glucosidase (which belongs to the GH family) activity is often a limiting factor in cellulase conversion and glucose release (Zang et al. 2018), the three compost-derived metagenomic libraries were analyzed through a functional screening performed on agar microplates containing esculin, the specific substrate for the β-glucosidase activity.

**Fig. 7:**
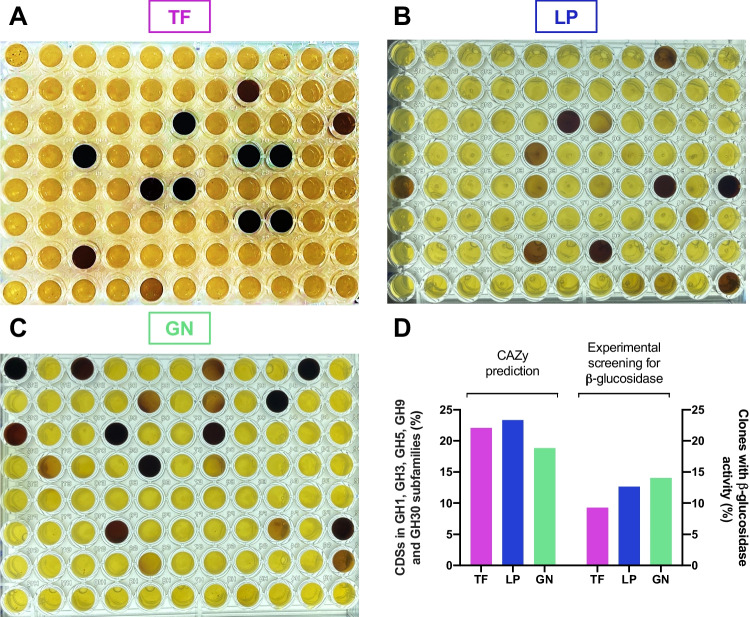
Experimental validation of β-glucosidase activity in the compost metagenomic libraries in comparison with β-glucosidase families predicted using the CAZy database. Visual representation of clones with positive response to the β-glucosidase activity for the three metagenomic libraries from **A** Terra Fértil (TF), **B** Lipor (LP), and **C** Gintegral (GN) compost samples. **D** Relative abundance of clones with β-glucosidase activity in the experimental screening and predicted CAZymes belonging to the GH1, GH3, GH5, GH9, and GH30 families in the TF, LP, and GN metagenomes

In this way, 563 clones from each metagenomic library were evaluated over 1 week and the observation of brown color was considered a positive response. All libraries exhibited a good number of positive clones for β-glucosidase activity, attesting the presence of these enzymes in the samples, in agreement with the sequencing approach. The metagenomic library constructed from the GN sample (Fig. 7C) presented a higher number of clones with β-glucosidase activity (79), followed by the LP library (71) and finally, the TF library with 59 positive clones (Fig. 7A and B). To compare the experimental data with the annotation against the CAZy database, the percentage of positive clones in each library was compared with the number of CDSs in the GH1, GH3, GH5, GH9, and GH30 families, which include β-glucosidases (Cairns and Esen 2010). Results show that compost metagenomes display around 19–22% of CAZymes belonging to the specified GH families, while 9–14% of clones were positive for β-glucosidase activity (Fig. 7D), effectively demonstrating the presence of these enzymes in the compost samples.

## Discussion

Exploring the aerobic composting ecosystem has been proved to be an interesting approach to find novel microorganisms and identify promising metabolites, namely, enzymes stable and active under harsh conditions. The composting samples studied in this work (TF, LP, and GN) presented high temperatures, since they were all collected specifically in the thermophilic phase of the composting process. The changes which occur at this phase are dependent on the growth and metabolism of thermophilic microorganisms, being affected by certain parameters, such as moisture, total carbon, and nitrogen contents. The significant differences obtained for these particular parameters between the TF and LP samples may be related with the presence of municipal sludges in the TF sample. Municipal sludges are rich in nitrogen and are characterized by high moisture content and low porosity (Kulikowska et al. 2022). Both, the TF and GN samples, presented a high percentage of municipal sludges and moisture content (> 63%). On the other hand, the moisture content (around 60%) recorded in the LP sample may be mainly associated with the inclusion of green and food residues which, in addition to the moisture, have also a high nitrogen content (Palaniveloo et al. 2020). The mixture of green organic wastes with lignocellulosic residues (brown residues) may have contributed to the differences observed in the total carbon and nitrogen contents. Green wastes generally present higher nitrogen content, while brown residues are mainly rich in carbon (Hemati et al. 2022; Kulikowska et al. 2022). For the three samples, the C to N ratios did not differ significantly, being obtained values around 11. Nevertheless, the samples studied in this work presented lower C to N ratios than those generally reported in the literature (Goyal et al. 2005; Awasthi et al. 2014; Chin et al. 2020). The lower C to N ratios together with the high moisture contents may explain the longer periods, due to a slower degradation, to reach the typical temperatures of the thermophilic phase of the composting process. Usually, temperatures above 50 °C are reached after a week (Guo et al. 2012; Awasthi et al. 2014; Zhang et al. 2020; Kulikowska et al. 2022). Regarding the pH variations, the TF and GN samples presented values close to 7 that benefit the microbial activities responsible for the decomposition of organic matter to produce organic acids and inorganic salts. The alkaline pH measured in the LP sample can be related to the type of residues (food wastes) that composed the sample, since an increase in pH is generally due to ammonia release as a consequence of protein degradation (Palaniveloo et al. 2020; Zhang et al. 2020). The humic acid content was significantly different for the three samples. The higher humic acid content in the LP sample can be related to a higher organic carbon content released from food wastes as they are more easily degraded than other recalcitrant materials. Also, the later sampling time of the LP sample (4 weeks) compared to the TF and GN samples can explain the amount of humic acids obtained, since these acids are generated over time according to the degradation of simpler components, followed by cellulose, hemicellulose, and lignin. On the other hand, the lower humic acid content in the GN sample may be associated with both the shorter sampling period and the sample composition. This sample was collected only after 2–3 weeks of the composting process, and it is known that humic acids can be used as substrate by thermophilic microorganisms in the early stages of the thermophilic phase (Unsal and Sozudogru Ok 2001; Hemati et al. 2022; Zhang et al. 2022), thus not being accumulated in the medium. Additionally, the GN sample presented a lower percentage of lignocellulosic materials and included cork chips in its composition, which is generally described as a very recalcitrant material for microbial degradation (Martins et al. 2014). All these facts might have affected the humic acid concentration obtained for the GN sample.

According to the literature, the process of organic matter degradation is essentially carried out by thermophilic microorganisms represented mainly by bacteria and fungi (Palaniveloo et al. 2020). In fact, our samples were clearly dominated by bacteria. The greater abundance of *Alphaproteobacteria* and *Gammaproteobacteria* (phylum *Proteobacteria*) was common for the three samples. The bacterial strains belonging to the phylum *Proteobacteria* are known to adapt to harsh ecosystems, namely, composting at the thermophilic phase (Zhou et al. 2017; Kaushal et al. 2018). The mentioned classes specifically harbor a wide variety of genes responsible for enhancing the degradation process of lignocellulose, as well as residual sludges, being the type of microorganisms commonly associated to the carbon and nitrogen cycles. In addition, they can break small molecules, such as glucose, propionate, and butyrate, and are positively related to neutral pH. All this evidence is in agreement with the characteristics of our samples. The low abundance of *Betaproteobacteria* in the TF and LP samples may explain the slower decomposition rate to reach high temperatures compared to the GN sample (Ma et al. 2020; Wang et al. 2020; Cao et al. 2021). Additionally, the two classes *Sphingobacteria* and *Flavobacteriia* (phylum *Bacteroidetes*) are also recognized for exhibiting a crucial role in the breakdown of recalcitrant polysaccharides in lignocellulose biomass (Le et al. 2022). The greater taxonomic difference was found in the TF sample, which presents in its composition an additional class, *Balneolia*, belonging to the phylum *Balneolaeota* (Costa et al. 2021). The members of this phylum are extremophiles capable of surviving and play an important role in extreme salt, temperature, or pH environments (Hahnke et al. 2016; Jo et al. 2021). The abundance of this class may be related to the higher temperature (62.7 °C) of the TF sample when compared to the LP and GN samples (50–52 °C), resulting in suitable conditions for the activity of this type of thermophilic microorganisms. However, other studies have reported the growth of microorganisms belonging to the *Balneolia* class in saline environments, namely, salterns and wastewater with high salinity (Jo et al. 2021; Almeida et al. 2022). This evidence may suggest that the residues used in the composting process of the TF sample, in particular municipal sludge, could present a salinity content that allows the development of this type of microorganisms.

Looking to the repertoire of MAGs, the taxonomic classification was very similar as all of them were classified into the *Bacteria* domain, confirming the results obtained before assembly. The abundance of *Gammaproteobacteria* and *Alphaproteobacteria* was also a common feature, as it was the presence of MAGs belonging to *Flavobacteriia*, *Sphingobacteriia*, and *Balneolia*, the latter only present in the TF sample. The large discrepancy between *Bacteria* and the other domains in terms of abundance and diversity of taxonomic units, both in the analysis of clean reads and MAGs, may arise not only from the sample characteristics but also from the chosen taxonomic database. Even though the kaiju nr_euk database was chosen as an attempt to characterize other microorganisms’ domains, most metagenomic databases include mainly bacterial data, or at least the most well-characterized non-bacterial taxa, so within the 30% of the unclassified reads, we need to account for the possible existence of less known and even novel non-bacterial taxa. Other databases and software were employed to expand the taxonomic characterization of the metagenomes, although with scarce results. Interestingly, the number of genomes that could be taxonomically classified at the species level was significantly decreased in all samples, suggesting that most MAGs herein reconstructed belong to undiscovered species, which require future endeavors to prompt their taxonomic classification. However, 7 species were assigned to the 9 MAGs and most of them were previously isolated from extreme environments, such as geothermal resources, *R. marinus* (Alfredson et al. 1988); deep-water/coastal areas, *P. indicus* (He et al. 2020), *G. marinus* (Li et al. 2013), and *B. sediminis* (Wang et al. 2015); and municipal solid waste landfills, *L. defluvii* (Yassin et al. 2007). In addition, the species *L. defluvii* (Hayat et al. 2013), *G. marinus* (Ballardo et al. 2020), *P. alcaliphilus* (Tian et al. 2013), *R. marinus* (Braga et al. 2021), and *P. gallinarii* (Song et al. 2015) are already related to biomass-degradation processes, namely, composting. These species have been recognized as crucial lignocellulose degraders given their ability to produce polysaccharide-degrading enzymes (e.g., cellulases, xylanases, and amylases) capable of acting under severe conditions, such as temperature and pH (Gomes et al. 2003; Hayat et al. 2013; Ballardo et al. 2020). These facts agree with the sampling temperature and pH of our samples. For instance, *P. alcaliphilus*, alkaliphilic bacteria (Urakami et al. 1989), was specifically found in the LP sample that differs from the TF and GN samples by its alkaline pH.

Consistent with the results (both using clean reads and MAGs) obtained in our study from COG and KEGG databases, the microbial functional profiles of the three samples confirm that the pathways associated with the metabolism of carbohydrates, energy, and amino acids are effectively the key pathways identified in any lignocellulosic composting, particularly in the thermophilic phase. During this phase of aerobic composting, a significant proportion of the microbial community acts on the decomposition of lignocellulose through carbohydrate and energy metabolisms. The fact that they are more active at this phase is due to the ability of microorganisms to quickly and easily degrade readily degradable carbohydrates from cellulose and hemicellulose (Kong et al. 2020; Zhang et al. 2020; Liu et al. 2022). In addition, the high abundance of metabolic functions of amino acids is triggered by the high metabolic intensity that characterizes the thermophilic phase, since amino acids provide the energy and carbon contents required for the growth and performance of microorganisms and can be used as feedstock for the synthesis of humic acids (Kong et al. 2020; Zhang et al. 2020, 2022; Li et al. 2021). The KEGG annotation also revealed a considerable number of genes associated with xenobiotic biodegradation and metabolism. The incidence of these complex compounds may result from the degradation of lignin at a later stage of the thermophilic phase (Zhang et al. 2022). In fact, the fast metabolism of amino acids, carbohydrates, and even lipids caused by high temperatures at this stage may have made available the energy and carbon content required to degrade the xenobiotic compounds (Kong et al. 2020).

The analysis of CAZymes present in our samples, considering both the clean reads and the MAGs, showed that the compost samples were enriched in GHs and GTs, as previously reported for other compost samples (Chang et al. 2022). Furthermore, the TF sample had a lower number of CDSs assigned as CAZymes, than the LP and GN samples. The same was observed when analyzing MAGs, since the LP and GN MAGs displayed a higher number of lignocellulolytic enzymes than the TF MAGs. GTs catalyze the formation of glycosidic bonds being involved in the biosynthesis of oligosaccharides, polysaccharides, and glycoconjugates (Breton et al. 2006). Among the different families, the GT2 family, which comprises enzymes derived from different sources and organisms that play a wide collection of functions (Breton et al. 2006), including cellulose/chitin synthase functions, is the most abundant. GT2 was also the main GT family represented in composting containing wheat straw, chicken manure, peanut meal, and gypsum, also in thermophilic conditions like our samples (Chang et al. 2022). However, in the context of lignocellulosic biomass conversion, GHs, which are responsible for the hydrolysis/transglycosylation of glycosidic bonds, are the most interesting enzymes as they present several catalytic activities including cellulases, endo-hemicellulases, debranching enzymes, and oligosaccharide-degrading enzymes that contribute to the composting process (Gong et al. 2020; Reichart et al. 2021). The GH13 family stood out in this study as being clearly the most abundant in the three samples. This family is the largest GH family and contains enzymes (e.g., hydrolases, transglycosidases, isomerases) exhibiting a broad range of substrate specificity and activities (Stam et al. 2006), from which the starch-degrading enzymes stand out for their potential for lignocellulose degradation (Gong et al. 2020). Other abundant GH families in the three samples were GH3, GH5, GH9, and GH31. Of note, GH3, GH5, and GH9 are among the main GH families responsible for lignocellulosic biomass degradation (Ezeilo et al. 2017), highlighting the great potential of the compost metagenomes herein presented for this process. Indeed, GH3 enzymes exhibit a vast diversity of hydrolytic activities (e.g., β-glucosidase, α-arabinofuranosidase, β-xylopyranosidase, and *N*-acetyl-β-glucosaminidase) that allow them to promote, among others, cellulosic biomass degradation and plant cell wall remodeling. GH5 and GH9 are important cellulase families that can act upon plant polysaccharides and carboxymethylcellulose, including endo- and exo-glucanases, endo- and exo-mannanases, β-glucosidases, and β-mannosidases (Ezeilo et al. 2017). Cellulases from the families GH5 and GH9 were the most represented in the analysis of MAGs. In a study that analyzed 60 MAGs recovered from two thermophilic composting cells, GH5 and GH9 were among the most represented cellulases in the top six degraders (Braga et al. 2021). GH5, GH8, and GH9 cellulose-degrading enzymes were also among the most abundant biocatalysts in the thermophilic compost studied by Chang et al. (2022).

The comparison study involving compost samples from the literature revealed a similar trend to the one obtained with our samples. The oligosaccharide-degrading enzymes from the GH3 family and the GH5 and GH9 cellulases were the most abundant in all cases, independently on the compost composition and process conditions, which attests again the great richness in lignocellulosic enzymes of composting environments. Importantly, the GHs’ percentage (Montella et al. 2017) and most abundant families (Allgaier et al. 2010; Kanokratana et al. 2018) previously reported were similar to those found in our study, proving the reliability of the pipeline herein implemented.

The taxonomic classification of the identified CAZymes was well aligned with the compost sample taxonomy as *Proteobacteria* and *Bacteroidetes* stood out as having the higher number of CDSs annotated as CAZymes. This evidence indicates that the taxonomic profile of the composting is directly related with its functional traits. An interesting abundance of CAZymes was also found in MAGs belonging to *Actinobacteria*, *Balneolaeota*, and *Deinococcus-Thermus*. Identification of CAZymes from *Proteobacteria*, *Bacteroidetes*, and *Actinobacteria* has been also reported in several composting samples such as those based on apple pomace (Zhou et al. 2017); on leaf and wood chip compost enriched in wheat straw, poplar, and *Miscanthus* (Heiss-Blanquet et al. 2016); and on shredded tree branches, leaves, grass, manure, beddings, and zoo animals’ food residues (Antunes et al. 2016). *Actinomycetales* appear to be common in the thermophilic and mature stages of the composting process, and CDSs annotated as lignocellulolytic enzymes belonging to this order have been reported before (Simmons et al. 2014; Antunes et al. 2016; Wang et al. 2016). Accordingly, a metatranscriptomic study on compost-derived microbial communities enriched on rice straw performed under thermophilic and mesophilic conditions revealed that lignocellulose-degrading enzymes mostly belonged to *Proteobacteria* and *Bacteroidetes* in the mesophilic microbial community, while the thermophilic was dominated by *Actinobacteria* (Simmons et al. 2014). Interestingly, members of *Proteobacteria* have also been found in samples retrieved in the end of the composting process, including *Enterobacteriales* and *Pseudomonadales*, contributing to the degradation of the remaining biomass (Antunes et al. 2016). CAZymes belonging to *Balneolaeota* were identified only in the TF sample. A *Balneolaeota* MAG predicted to have over 10 GH13 genes was recovered from a saltern pond sample with 7.5% salinity (Kimbrel et al. 2018), reinforcing again the possibility of the higher salinity of this sample in comparison with the other two under study. Lignocellulose-degrading enzymes were also found in a *Deinococcus-Thermus* MAG recovered from the GN sample. β-Glucosidases belonging to this phylum were also found in cow manure and rice straw composting at the thermophilic phase, which is in good agreement with the extremophile nature of the members of this phylum (Zang et al. 2017).

The experimental screening for β-glucosidase activity validated our bioinformatic pipeline as it confirmed the great abundance of positive clones exhibiting this catalytic activity, generally associated to enzymes belonging to the GH1, GH3, GH5, GH9, and GH30 families (Cairns and Esen 2010). Indeed, our samples showed great abundance of GHs within CAZymes, specifically the GH3, GH5, and GH9 families, as aforementioned. Since β-glucosidases are generally the rate-limiting enzymes in the cellulose degradation (Zang et al. 2018), the identified enzymes, which are likely to be active at high temperatures considering the thermophilic origin of the compost, can represent a promising enzymatic pool to be explored for cellulose degradation in plant biomass under harsh industrial conditions. The lower percentage obtained in the experimental approach in comparison with the bioinformatic prediction may be explained by the need for optimizing the growth conditions, the catalytic activity, and, probably, the host itself. In fact, the screening was performed in standard conditions of temperature and incubation times, and further optimization may render a similar percentage to the ones predicted in silico.

In conclusion, the taxonomy and functional profile of the compost samples’ microbiome was successfully characterized using the developed bioinformatic pipeline, which was validated using previously reported data. Bacteria clearly dominated all samples, with the classes *Gammaproteobacteria*, *Alphaproteobacteria*, and *Balneolia* being the most abundant. These data suggest that bacterial enzymatic activity is the main driver of lignocellulose degradation in the thermophilic compost samples herein studied. The analysis of our samples together with samples retrieved from the literature confirmed that composting, independently of the composition and process conditions, is a promising source of lignocellulolytic enzymes, especially GHs, which goes well in line with one of the most abundant functional categories predicted in our samples (carbohydrate transport and metabolism). Indeed, around 1% of the CDSs of the compost metagenomes were predicted to be from putative CAZymes. Specifically within GHs, our samples were enriched in cellulases from the GH5 and GH9 families and oligosaccharide-degrading enzymes from the GH3 family. These families are known to contain β-glucosidases, whose catalytic activity was further validated by our experimental functional screening. As our samples were retrieved from the thermophilic phase of the composting process, it is expected that at least a considerable proportion of the identified bacterial GHs would be resistant to harsh conditions, namely, high temperatures. Therefore, they could be considered promising biocatalysts for developing efficient and sustainable strategies for lignocellulosic biomass conversion.

## Supplementary information

Below is the link to the electronic supplementary material.

Supplementary file1 (XLSX 2752 KB)

## Data Availability

The authors confirm that the datasets supporting the findings and conclusions of this study are available within the article and its supplemental information file. Additional data can be provided upon request.
